# University Students’ Good Practices as Moderators Between Active Coping and Stress Responses

**DOI:** 10.3390/bs15091223

**Published:** 2025-09-09

**Authors:** Cristina Ruiz-Camacho, Margarita Gozalo, Elena Felipe-Castaño

**Affiliations:** 1 Department of Psychology and Anthropology, Faculty of Education and Psychology, University of Extremadura, 06071 Badajoz, Spain; cristinarc@unex.es; 2 Department of Psychology and Anthropology, Faculty of Sport Science (Psychology Laboratory), University of Extremadura, 10005 Cáceres, Spain; 3 Department of Psychology and Anthropology, Faculty of Teacher Training College, University of Extremadura, 10071 Cáceres, Spain; efelipe@unex.es

**Keywords:** good practices, active coping, stress responses, university students, higher education, moderation

## Abstract

Within the framework of the European Higher Education Area, university students’ good practices are considered key indicators of educational quality. In light of the high levels of academic stress reported in this population, the present study aims to examine whether four specific practices—feedback-seeking, cooperative work, time management, and active learning—moderate the relationship between active coping and stress responses. A cross-sectional design was employed with a sample of 1014 university students (*M* = 20.56; *SD* = 3.50). Participants completed the Academic Stress Coping Scale (A-CEA), the Academic Stress Response Scale (R-CEA), and the Inventory of Good Practices in University Students (IBPEU). Moderation analyses were conducted using linear regressions with interaction terms, and conditional effects (simple slopes) were estimated at low and high levels of the moderator. Significant moderation effects emerged. Feedback-seeking, cooperative work, and time management strengthened the inverse association between active coping and academic stress, with stronger reductions when these practices were reported at high levels. In contrast, active learning showed a threshold pattern: active coping reduced stress only when this practice was actively implemented, suggesting that its effective implementation may be necessary for coping to be effective. Promoting good practices may enhance the benefits of active coping. Their integration into early psychoeducational programs could bolster students’ personal resources and reduce psychological distress in demanding academic settings.

## 1. Introduction

In recent years, there has been a sustained decline in psychological well-being among university students, with academic stress emerging as one of the most prevalent and persistent manifestations (American College Health Association, 2023; Lipson et al., 2019). Recent data confirm that the majority of students report moderate to high levels of academic stress, with a clear trend toward severe levels (Bezie et al., 2025; Kaphle et al., 2024).

This psychological arises within a particularly demanding context, where multiple sources of tension converge: evaluative pressure, academic overload, diverse pedagogical methodologies, and the frequent need to balance studies with work, social, or financial responsibilities (Jirjees et al., 2024; Olson et al., 2025). While a certain level of arousal stemming from academic demands may foster engagement and enhance performance (You, 2018), sustained exposure to such demands leads to a state of chronic stress associated with physical, emotional, and behavioral manifestations (e.g., sleep problems, fatigue, irritability, somatic complaints, substance use, and sedentary lifestyles), which may further impair students’ psychological and academic adjustment (AlHamlan et al., 2025; Benítez-Agudelo et al., 2025; Bulfone et al., 2025).

Nevertheless, understanding academic stress requires moving beyond its conception as an individual reaction to study demands (Peeters et al., 2025) and recognizing the structural and organizational components of the university system as integral to the stress experience (Román & Hernández, 2011). Thus, the key issue is not only how students cope with demands but also which educational practices are fostered within the university context—such as planning study, integrating feedback, engaging in cooperative dynamics, or adopting active learning approaches—as these strategies may enhance the effectiveness of coping (Li & Han, 2024; Wong & Lai, 2025).

### 1.1. Coping from Transactional and Systemic-Cognitive Perspectives

Coping constitutes a central process in the experience of academic stress. According to Lazarus and Folkman (1984), stress does not depend exclusively on the objective characteristics of a situation, but rather on the individual’s subjective appraisal of their resources to manage it. This cognitive appraisal process—comprising both primary and secondary evaluations—determines whether the situation is perceived as threatening and whether the person believes they possess sufficient resources to deal with it. From this transactional perspective, coping refers to a set of cognitive and behavioral efforts aimed at managing stressful demands. These strategies may focus on either the emotion or the problem, depending on the nature of the situation and the individual’s perception. Problem solving, cognitive restructuring, and seeking social support are among the most common forms of active coping (González Cabanach et al., 2018; Valdivieso-León et al., 2020), and their effectiveness has been linked to lower perceived stressors, greater psychological well-being, and improved academic performance (Freire et al., 2020; Gustems-Carnicer et al., 2019a).

In a complementary line, the systemic-cognitive model proposed by Barraza (2006, 2018) expands the transactional perspective by conceptualizing academic stress as the result of an ongoing interaction between perceived demands and the student’s available resources to cope with them, within a dynamic person–environment relational system. This model places coping as a key self-regulatory mechanism aimed at restoring systemic balance when a functional mismatch occurs. From this viewpoint, imbalance triggers adaptive processes that involve both individual strategies and contextually attuned responses.

### 1.2. Students’ Good Practices in the University Ecosystem

Muñoz (2004) underscores the importance of organizational factors within the university system as key components shaping student well-being. His analysis points to shortcomings in curricular and program planning, the excessive demands created by overlapping schedules and coursework, and the limited involvement of students in related decision-making processes. In turn, Román and Hernández (2011) highlight the absence of institutional and didactic adjustment mechanisms capable of addressing the structural sources of stress, beyond isolated interventions directed at the individual.

Amid ongoing transformations within the European Higher Education Area (EHEA), universities have increasingly embraced a quality-oriented culture centered on student needs. This shift has fostered the adoption of educational models that promote active engagement, shared responsibility in learning, and students’ holistic development (Fraile et al., 2023; Gozalo Delgado et al., 2022). In this evolving context, identifying and fostering university students’ good practices has gained particular importance. These practices are conceptualized as a set of personal, relational, and situational resources that shape how students cope with academic demands and navigate the life transitions inherent in the university experience (Gozalo Delgado & León del Barco, 2014; Schlossberg et al., 1995).

From this perspective, good practices go beyond learning styles, encompassing both intrapersonal and interpersonal skills that can be nurtured and developed within the university context (Pinheiro, 2008). In this regard, Chickering and Gamson (1987) argued that quality education is grounded in principles such as student–faculty interaction, peer collaboration, active learning, ongoing feedback, effective time management, the communication of high expectations, and respect for diverse approaches to learning. Over time, these principles have been consolidated as a foundational reference for the subsequent conceptualization of good practices in higher education.

### 1.3. Articulation Between Good Practices and Coping with Academic Stress

Specific academic practices adopted by university students have been consistently linked to higher levels of self-regulation and more effective adaptation to academic demands. For instance, time planning and organization are associated with increased academic productivity, greater self-control, and lower stress perception (Fu et al., 2025; Patzak et al., 2025). The deliberate use of feedback strengthens self-efficacy and contributes to more accurate metacognitive monitoring of one’s learning process (Huisman et al., 2019; Young & Carless, 2024). Cooperative work, when structured around shared responsibility, fosters co-regulation and task engagement, yielding positive effects on both cognitive and emotional outcomes (Chen et al., 2025; Greisel et al., 2024). Lastly, active learning entails meaningful involvement in the construction of knowledge, encouraging a proactive and self-regulated learning approach (Bintz et al., 2024; Diningrat et al., 2024). Collectively, these practices represent intentional processes of managing one’s learning and academic environment, thus playing a pivotal role in students’ adjustment and adaptation to university life.

Its adaptive value can be examined through the lens of the coping framework proposed by Aspinwall and Taylor (1997), who define coping not merely as a preferred strategy but as a set of developable skills—including goal setting, planning, organization, and the analysis of complex situations. From this perspective, university students’ good practices may be conceptualized as operational expressions of such skills, with the potential to shape how academic demands are addressed. Along these lines, Gustems-Carnicer et al. (2019b) argue that possessing functional coping resources serves as a powerful buffer in high-pressure and threat-laden contexts, helping to mitigate their impact on psychological well-being.

### 1.4. The Present Study

Despite the growing interest in university students’ good practices, empirical evidence regarding their role in academic stress remains limited, particularly with respect to their capacity to modulate the relationship between coping strategies and stress-related responses. The present study aims to advance this line of research by examining whether four specific practices—feedback-seeking, cooperative work, time management, and active learning—play a role in this dynamic. This approach aligns with the Self-Regulated versus Externally Regulated Learning (SRL vs. ERL) model (De la Fuente-Arias, 2017), which posits that personal self-regulation is more effective when it is supported by regulatory elements within the educational environment.

We therefore begin with the premise that, in contexts where these practices are more prevalent, active coping will exhibit a stronger negative association with academic stress responses. This general premise underlies the composite hypothesis that guides the study.

**Figure 1:**
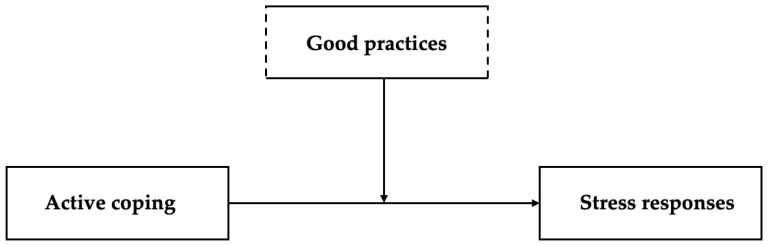
Hypothesized model.

**H1.**  *University students’ good practices—feedback-seeking (**H1a**), cooperative work (**H1b**), time management (**H1c**), and active learning (**H1d**)—will act as moderating variables in the relationship between active coping and academic stress responses, enhancing the protective effect of coping on psychological distress (Figure 1).*

## 2. Methodology

### 2.1. Design and Participants

This study employed a quantitative, descriptive, and non-experimental methodology, using a cross-sectional ex post facto design (Ato et al., 2013).

Data collection took place at the University of Extremadura (UEx), a public Spanish institution with main campuses in Badajoz, Cáceres, Mérida, and Plasencia. Based on institutional records, UEx has approximately 24,000 students enrolled in official undergraduate and postgraduate programs, in addition to around 8000 students registered in doctoral studies, continuing education, and other non-degree programs.

The study focused on students enrolled in on-site undergraduate programs, with the reference population estimated at approximately 18,000 during the 2024/2025 academic year, according to institutional data. Assuming a 95% confidence level, a 3% margin of error, and an expected proportion of 50%—a conservative estimate maximizing variance—the minimum required sample size was calculated at 1008 participants. In total, 1014 valid responses were obtained following questionnaire administration.

The final sample comprised students aged between 17 and 63 years (*M* = 20.56, *SD* = 3.50). Of these, 64.5% were women (*n* = 654) and 35.5% were men (*n* = 360). Distribution by academic year was as follows: second year (28.8%, *n* = 292), first year (27.9%, *n* = 283), fourth year (23.3%, *n* = 236), and third year (20.0%, *n* = 203). With regard to fields of study, the largest group was from Social and Legal Sciences (43%, *n* = 436), followed by Health Sciences (22.6%, *n* = 229), Sciences (17.2%, *n* = 174), Engineering and Architecture (8.8%, *n* = 89), and Arts and Humanities (8.5%, *n* = 86).

Inclusion criteria required students to be enrolled in an official undergraduate program at the University of Extremadura during the 2024/2025 academic year and to voluntarily consent to participate by signing the informed consent form. Although the study was primarily targeted at students over the age of 18, participation was also allowed for first-year students under the age of majority, provided they met the above criteria and that all ethical principles related to research with minors were strictly upheld.

### 2.2. Measures

#### 2.2.1. Active Coping

This variable was assessed using the Academic Stress Coping Scale (A-CEA; R. G. Cabanach et al., 2010), which examines the active coping strategies employed by university students to manage stressful academic situations. The instrument comprises 23 items rated on a 5-point Likert scale (1 = “never” to 5 = “always”). It evaluates behaviors such as constructively reinterpreting stressful events, seeking advice or support from others, and planning academic tasks in advance. Example items include: “*When faced with a problematic situation, I forget the unpleasant aspects and focus on the positive*” and “*I ask for advice from a family member or a close friend*”. In the present study, the scale showed excellent internal consistency, with a cronbach’s alpha of 0.91.

#### 2.2.2. Stress Responses

This variable was assessed using the Academic Stress Response Scale (R-CEA; R. Cabanach et al., 2008), which measures the frequency with which students experience symptoms associated with academic stress. The instrument consists of 22 items that assess indicators across three levels of response: physiological, cognitive, and behavioral. Responses are recorded on a 5-point Likert scale (1 = “never” to 5 = “always”). Example items include: “*In recent weeks, any minor inconvenience irritates me*” and “*In recent weeks, I tend to highlight my failures and downplay my successes*”. In the present study, the scale demonstrated excellent internal consistency, with a Cronbach’s alpha of 0.93.

#### 2.2.3. University Students’ Good Practices

These were assessed using selected subscales from the Inventory of Good Practices of University Students (IBPEU; Chickering & Schlossberg, 1995; Spanish version adapted by Gozalo Delgado et al., 2012). This instrument evaluates how frequently students engage in behaviors and attitudes consistent with the core principles of good practices in higher education. It comprises 9 independent subscales of 7 items each, rated on a 5-point Likert scale (1 = “never” to 5 = “always”). In the present study, four of these subscales were analyzed, selected for their stronger theoretical relevance to the management of academic stress (Chen et al., 2025; Patzak et al., 2025). In addition, a preliminary exploratory analysis, conducted by the present research team with the same university population, indicated that these were the subscales showing the most consistent associations with stress indicators, which supports their selection. The following sections describe them.

(a) Feedback-Seeking

This subscale assesses the extent to which students actively seek, value, and use feedback from both faculty and peers to improve their academic performance. It reflects a proactive attitude toward reviewing, refining, and adjusting one’s learning strategies. Sample items include: “*I revise my work when necessary and seek feedback from my instructors to do so*” and “*I appreciate feedback from my peers and take it into account in my actions.*” The cronbach’s alpha obtained in the present study was 0.95.

(b) Cooperative Work

This subscale assesses students’ involvement in collaborative academic activities with their peers, both inside and outside the classroom. It refers to behaviors that foster shared learning, mutual support, and the co-construction of knowledge. Sample items include: “*Outside the classroom, I study or work in groups with other students*” and “*I help my classmates when they ask for assistance.*” The cronbach’s alpha obtained in the present study was 0.74.

(c) Time Management

This subscale captures students’ efficient use of time in relation to academic demands. It assesses their ability to organize, plan, and allocate study time effectively in order to meet deadlines and maintain consistent progress in their coursework. Sample items include: “*I complete assignments within the established deadlines*” and “*I schedule my study sessions to keep up with my courses.*” The cronbach’s alpha obtained in the present study was 0.71.

(d) Active Learning

This subscale evaluates students’ involvement in their own learning process, emphasizing their initiative to expand, apply, or deepen course content beyond classroom requirements. It reflects a proactive attitude aimed at constructing knowledge from an autonomous and meaningful perspective. Sample items include: “*I look for new readings and/or research projects related to my courses and/or my degree*” and “*I try to relate everyday events and activities to the topics discussed in class.*” The cronbach’s alpha obtained in the present study was 0.73.

#### 2.2.4. Control Variables

Two control variables were included, collected through a brief sociodemographic questionnaire, in order to statistically adjust for potential confounding effects. Gender was coded as a dichotomous variable (0 = male; 1 = female), and age was recorded as a continuous variable, expressed in years.

### 2.3. Procedure

After obtaining the necessary authorizations from the university departments, the research team accessed various undergraduate classrooms at the University of Extremadura across different academic disciplines. Data collection took place during regular class hours, with participation organized through class groups and with the approval of the teaching staff. In this context, students were invited to voluntarily participate in the study and were provided with clear information about its objectives and procedures. Upon giving their informed consent digitally, participants completed the questionnaires, which were compiled into a single online form.

Access to the form was provided via a QR code, allowing students to complete it individually through the Google Forms platform. The estimated completion time was approximately 20 min. Participants were informed that the data would be used exclusively for scientific purposes and were reminded that there were no right or wrong answers, to reduce social desirability bias. Additionally, the researchers were present to address any questions that arose during the process.

Several methodological precautions were taken to ensure data quality: all form fields were mandatory, so no incomplete responses were recorded. After data collection, responses were reviewed to identify potential inconsistencies or out-of-range values. The survey was administered during the second semester of the 2024/2025 academic year, avoiding exam periods to minimize potential bias related to academic evaluation. The authors of the manuscript are teaching and research staff at the university. The study was conducted in accordance with the principles of the Declaration of Helsinki and the Ethical Code of the UEx.

### 2.4. Data Analysis

Preliminary analyses were conducted, including summary statistics and correlation matrices to explore relationships between the main study variables.

Subsequently, regression models with interaction effects were applied to examine whether university students’ good practices moderated the relationship between adaptive coping and academic stress responses. A bootstrapping procedure with 1000 samples was employed, using 95% confidence intervals. In all models, gender and age were included as control variables. The assumptions of normality, independence, and homoscedasticity of the residuals were tested using the Kolmogorov–Smirnov, Durbin–Watson, and Levene’s tests, respectively.

For significant interactions, simple slope analyses were conducted to examine the effect of active coping on academic stress responses at low and high levels of each moderator. All analyses were performed using SPSS software (version 26.0) and the PROCESS macro (version 3.4), adopting a significance level of *p* ≤ 0.05.

## 3. Results

### 3.1. Initial Analyses

Means, standard deviations, and bivariate correlations are presented in Table 1. Active coping was significantly associated with lower levels of stress responses (*r* = −0.27, *p* < 0.01). Moreover, the R-CEA scale exhibited negative correlations with all dimensions of good practices, except for active learning, which showed a positive association (*r* = 0.23, *p* < 0.01). The strongest correlation emerged with the time management dimension (*r* = −0.41, *p* < 0.01).

### 3.2. Regression Analysis with Interaction Effects

#### 3.2.1. Moderation Analysis One: Feedback-Seeking

Moderation analysis (Table 2) revealed that both active coping and feedback-seeking exhibited significant negative effects on stress responses, indicating that higher levels of coping and feedback-seeking were associated with lower levels of academic distress. In addition, a significant interaction effect between these two variables was identified (*B* = −0.38, *p* = 0.032, 95% CI [−0.21, −0.01]), suggesting a moderating role of feedback-seeking. The model was statistically significant (*F*(1008, 1013) = 21.72, *p* < 0.001), accounted for 19.7% of the variance, and met the assumptions of normality, independence, and homoscedasticity. Finally, gender was significantly associated with stress responses (*B* = 0.22, *p* < 0.001).

**Figure 2:**
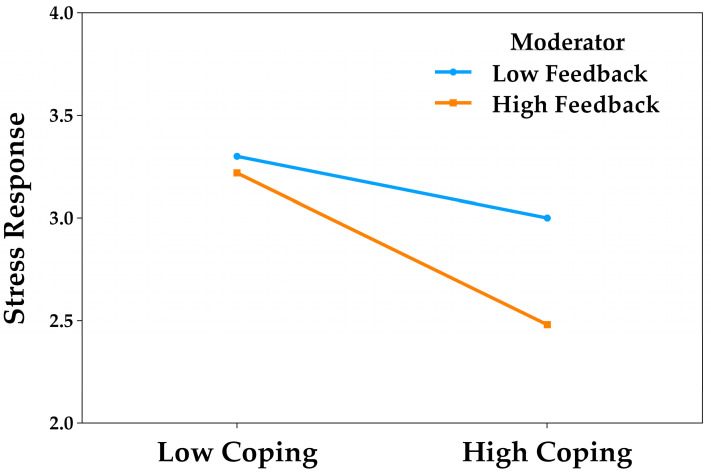
Moderating effect of feedback-seeking in the relationship between active coping and stress responses.

The simple slopes analysis revealed that active coping significantly predicted a reduction in stress responses both when feedback-seeking was low (*B* = −0.15, *t* = −2.10, *p* = 0.036, 95% CI [−0.29, −0.01]) and when it was high (*B* = −0.37, *t* = −3.45, *p* = 0.001, 95% CI [−0.57, −0.16]). In other words, as coping increased, stress responses decreased, with this effect being more pronounced in the group with high levels of feedback-seeking. This pattern suggests that this academic good practice enhances the protective effect of coping on stress. Figure 2 illustrates this interaction.

#### 3.2.2. Moderation Analysis Two: Cooperative Work

As shown in Table 3, both active coping and cooperative work significantly predicted lower levels of stress responses. A significant interaction effect between the two variables was also observed (*B* = −0.17, *p* = 0.038, 95% CI [−0.33, −0.01]), indicating that cooperative work moderated the relationship between coping and academic stress. The model was statistically significant (*F*(1008, 1013) = 21.22, *p* < 0.001), explained 18.5% of the variance, and met the assumptions of normality, independence, and homoscedasticity. Gender was again significantly associated with stress responses (*B* = 0.22, *p* < 0.001).

**Figure 3:**
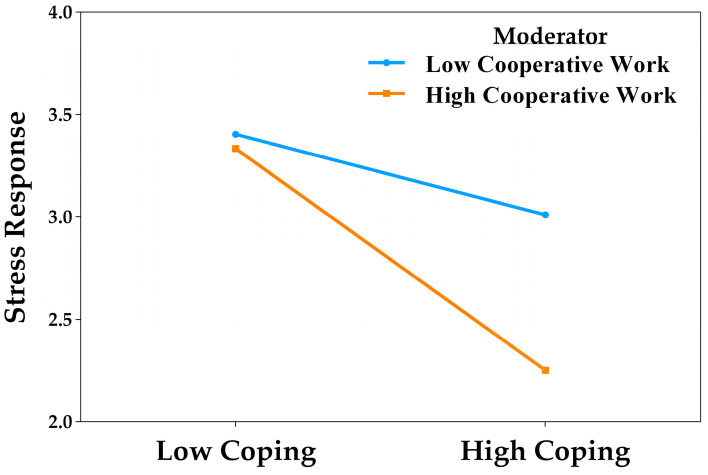
Moderating effect of cooperative work in the relationship between active coping and stress responses.

The simple slopes analysis showed that active coping significantly predicted a reduction in stress responses both under low (*B* = −0.20, *t* = −2.12, *p* = 0.035, 95% CI [−0.39, −0.01]) and high levels of cooperative work (*B* = −0.54, *t* = −4.10, *p* < 0.001, 95% CI [−0.78, −0.29]). In both cases, higher coping levels were associated with lower stress responses, with this effect being more pronounced among students who were highly engaged in cooperative work. These results suggest that this academic practice strengthens the protective role of coping. The interaction is depicted in Figure 3.

#### 3.2.3. Moderation Analysis Three: Time Management

Moderation analysis (Table 4) revealed significant negative effects of both active coping and time management on stress responses, suggesting that higher levels of coping and effective time use were associated with lower academic distress. A significant interaction effect was identified between the two variables (*B* = −0.18, *p* = 0.026, 95% CI [−0.34, −0.02]), supporting the presence of a moderating effect. The model was statistically significant (*F*(1008, 1013) = 26.43, *p* < 0.001), accounted for 21.6% of the variance, and met all assumptions of normality, independence, and homoscedasticity. Gender remained a significant predictor of stress responses (*B* = 0.27, *p* < 0.001).

**Figure 4:**
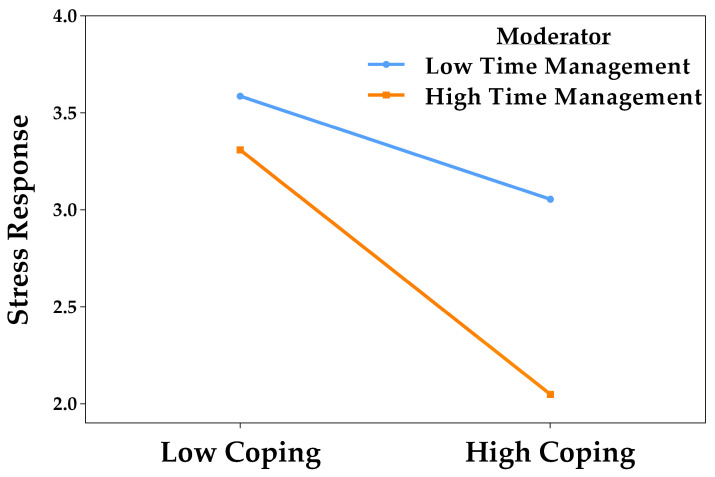
Moderating effect of time management in the relationship between active coping and stress responses.

The simple slopes analysis showed that active coping significantly predicted a reduction in stress responses both when time management was low (*B* = −0.27, *t* = −2.22, *p* = 0.027) and high (*B* = −0.63, *t* = −3.20, *p* = 0.001). As coping increased, stress decreased. This effect was stronger among students with high levels of time optimization, suggesting that effective time management amplifies the stress-reducing effect of coping. Figure 4 represents this interaction.

#### 3.2.4. Moderation Analysis Four: Active Learning

In the moderation analysis related to active learning (Table 5), significant effects were found for both active coping and this good practice dimension. Specifically, coping was negatively associated with stress responses (*B* = −0.41, *p* < 0.001, 95% CI [−0.63, −0.19]), whereas active learning showed a positive association (*B* = 0.32, *p* = 0.002, 95% CI [0.10, 0.53]). A significant interaction effect was also identified (*B* = −0.23, *p* = 0.019, 95% CI [−0.43, −0.03]), indicating a moderating effect. The model was statistically significant (*F*(1008, 1013) = 22.55, *p* < 0.001), explained 23.1% of the variance, and satisfied the assumptions of normality, independence, and homoscedasticity. Gender again showed a significant association with stress responses (*B* = 0.21, *p* < 0.001).

**Figure 5:**
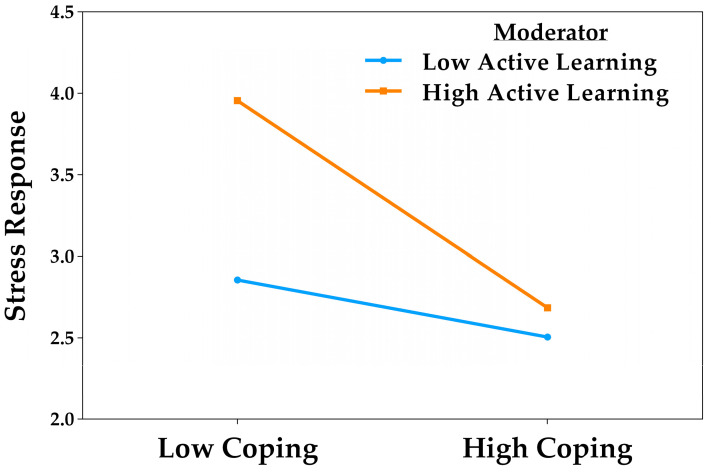
Moderating effect of active learning in the relationship between active coping and stress responses.

The simple slopes analysis showed that active coping significantly predicted a reduction in stress responses only when active learning levels were high (*B* = −0.64, *t* = −4.31, *p* < 0.001, 95% CI [−0.93, −0.35]). In contrast, this relationship was not significant when active learning was low (*B* = −0.18, *t* = −1.21, *p* = 0.226, 95% CI [−0.47, 0.11]). In other words, coping only proved effective in reducing stress when accompanied by a high level of active learning. This interaction, illustrated in Figure 5, supports the presence of a moderation effect.

Taken together, the moderation analyses showed that students’ good practices reinforced the protective effect of active coping against academic stress. Feedback-seeking, cooperative work, and time management acted as consistent facilitators: at both low and high levels of these practices, coping was associated with reduced stress, although the magnitude of the effect was greater at higher levels. Active learning, by contrast, exhibited a distinct pattern. Directly, this dimension was positively associated with stress responses, suggesting that greater student involvement in active learning strategies or activities may heighten academic distress. However, the interaction analysis revealed a threshold effect: coping reduced stress only when active learning reached high levels. In other words, while active learning on its own may be linked to increased distress, its combination with effective coping strategies transforms this relationship and enhances the capacity of coping to mitigate stress.

## 4. Discussion

The main objective of this study was to examine the extent to which specific good practices among university students—namely, seeking feedback, engaging in cooperative work, optimizing the time devoted to academic tasks, and active learning—moderate the relationship between active coping and academic stress responses. Based on the premise that such practices function as contextual resources that enhance the protective efficacy of coping, four sub-hypotheses (H1a–H1d) were proposed, each aimed at analyzing the moderating effect of a specific practice.

The results consistently supported the general hypothesis. In all four cases analyzed, statistically significant interaction effects were identified, even after controlling for the potential influence of sociodemographic variables (age and gender). Overall, the models accounted for between 18% and 23% of the variance in academic stress responses, suggesting a substantial contribution of good practices in modulating the impact of active coping.

However, the nature of the interaction varied depending on the specific dimension of good practices considered. The following sections discuss the results for each of them, with a focus on the differential implications arising from their interaction with coping.

In the case of feedback-seeking, active coping was associated with a significant reduction in academic stress at both low and high levels of this practice. However, the decrease was substantially more pronounced when feedback seeking was high. This pattern suggests that this dimension functions as a contextual facilitator by enhancing the regulatory impact of coping strategies. This finding aligns with previous studies highlighting the dual role of feedback as both an instrumental resource—by providing specific information to adjust learning strategies—and an affective one, by offering validation, guidance, and emotional support (Henderson et al., 2021; Khuder, 2025). In this regard, our results reinforce the notion that academic feedback, when actively sought and valued by students, can increase the effectiveness of personal coping resources in high-demand contexts by supporting both cognitive and emotional self-regulation (Jeffery & Halcomb-Smith, 2020).

A similar dynamic was observed for the cooperative work dimension. Active coping was associated with a significant reduction in academic stress across all levels of group involvement, although this effect was notably stronger among students who reported high participation in collaborative activities. This pattern suggests that peer collaboration functions as a facilitating social condition by enhancing the effectiveness of coping strategies in academically demanding contexts. Horizontal collaboration not only provides academic support but also strengthens key socioemotional resources, fosters mutual validation, and contributes to the consolidation of adaptive responses to institutional pressure (Backer et al., 2015; Huang et al., 2024; Liu & Ye, 2025). While its impact on academic performance and motivation has been traditionally emphasized, the findings of the present study extend its relevance by highlighting its specific role in the regulation of academic stress. In line with this, findings from longitudinal studies support that cooperative learning enhances peer relationships, reduces stress, and fosters student engagement in contexts of high academic pressure (Van Ryzin & Roseth, 2021).

Time management significantly enhanced the effect of active coping, such that students who reported more efficient temporal organization experienced a markedly greater reduction in academic stress compared to those with lower levels of this practice. Effective time use not only frees up attentional resources but also reduces perceived pressure and fosters a stronger sense of control over academic demands (Abojedi et al., 2023; Ahmady et al., 2021; Chust-Hernández et al., 2024). In this regard, time management operates as a structural support that amplifies the protective role of coping strategies in mitigating academic distress.

Active learning displayed a differentiated pattern compared to the other good practices analyzed. Considered in isolation, it was associated with higher levels of stress responses, consistent with studies reporting a transient increase in discomfort when active methodologies require substantial involvement, autonomy, and responsibility, particularly in the absence of adequate self-regulatory support (Brigati et al., 2020; Cooper et al., 2018; England et al., 2019). In our study, stress reduction occurred only when high levels of coping coincided with high levels of active learning. Conversely, when coping was low, greater active engagement did not exert a protective effect and was even associated with increased distress. Thus, in contexts that combine active learning with mechanisms designed to foster self-regulation, this dimension may operate as a catalyst for adaptive coping, promoting a more balanced academic experience (Bingen et al., 2019; De la Fuente et al., 2020; Wang et al., 2022).

Based on these findings, two distinct moderation profiles were identified. On the one hand, feedback-seeking, cooperative work, and time management operated as amplifying practices: active coping was associated with lower academic stress across the sample, but this association was notably stronger when these practices were reported at high levels. On the other hand, active learning followed a threshold pattern: coping was effective in reducing stress only among students with high levels of active engagement. This suggests that such pedagogical approaches demand solid self-regulatory competencies to activate their protective potential without becoming an additional stressor. Gender showed a small residual effect, with women reporting slightly higher stress levels, although it did not alter the direction or strength of the observed relationships. Overall, the results highlight that active coping does not operate in isolation; rather, its effectiveness is contingent upon the presence of specific good practices that, to varying degrees, facilitate its enactment in demanding academic contexts.

### 4.1. Theoretical and Practical Implications

From a theoretical standpoint, the findings support the SRL vs. ERL model (De la Fuente-Arias, 2017), demonstrating that the effectiveness of active coping—as an indicator of internal self-regulation—depends on the presence of academic practices that function as external supports: seeking feedback, cooperative work, time management, and active learning. At the same time, they align with the psychoeducational perspective of Román and Hernández (2011), which underscores the need to incorporate didactic and organizational variables into the analysis of academic stress. From this perspective, good student practices go beyond individual strategies to become structural adjustment mechanisms capable of modulating the impact of university demands. Overall, the results shift the focus from a purely psychological interpretation of stress toward a broader ecological framework, in which feedback culture, time organization, and methodological choices emerge as key components for student well-being.

In terms of practical implications, the results provide a clear framework for university-level interventions, reinforcing the idea that good student practices can be deliberately fostered to enhance the protective value of active coping. Three complementary levels of action are proposed, in line with the Health Promoting University framework (Sáenz et al., 2025), which integrates institutional policies, organizational culture, and community well-being for the benefit of both students and faculty.

(1) Low-cost, high-impact transversal measures. From the first semester and throughout the initial stages of university study, it is recommended to implement rubrics with explicit criteria and formative feedback on academic performance, establish virtual forums for timely consultation on tasks and content, and provide targeted short-format workshops on time management (Chust-Hernández et al., 2024). These measures strengthen feedback, peer collaboration, and time management—three practices identified in our study as progressive enhancers of active coping.
(2) Scaffolded pathways for active learning with self-regulatory support. Active methodologies—such as project-based learning or flipped classrooms—can be designed to incorporate structured opportunities for goal setting, periodic self-assessment, and training in coping strategies. These learning environments should progressively scale task complexity and integrate guided regulation tools such as checklists or debriefing sessions (Bingen et al., 2019; Nicol & Macfarlane-Dick, 2006). Such scaffolding is critical, as our study revealed a threshold effect: active learning only reduced stress when combined with high engagement and consolidated self-regulatory skills. At this level, it is also particularly pertinent to integrate well-established cooperative learning models, such as Project-Based Collaborative Learning (PBCL), which effectively combines project-based problem solving with collective group responsibility and shared socio-emotional regulation (Huang et al., 2024). Likewise, cooperative learning strategies and peer tutoring methods (Durán Gisbert et al., 2015) have proven effective in both enhancing academic achievement and fostering socio-emotional competencies.
(3) Institutional policies for academic well-being. At the organizational level, it is important to incorporate indicators of good practices into teaching quality assurance systems—for example, the frequency and perceived usefulness of feedback provided and requested (Khuder, 2025). In addition, systematically assessing these practices among students would allow the identification of profiles of strengths and areas for improvement, offering a dynamic view of how they cope with academic stress. This information could be integrated into tutorial action plans and student support services, facilitating preventive and personalized interventions.

### 4.2. Limitations and Future Research Directions

While acknowledging their significance, the findings of this study must be interpreted considering certain limitations. Primarily, the cross-sectional design precludes causal inference and constrains the examination of the temporal dynamics of the variables under study. Longitudinal methodologies would provide a more robust framework to analyze the development and stability of these associations over time. Secondly, a non-probability cluster sampling procedure was employed, which limits representativeness and advises caution in the generalization of the results. Third, reliance on self-reported questionnaires may have introduced biases related to social desirability or subjective interpretation.

Moreover, the good practices assessed were not the result of a specific intervention, nor were they actively trained; instead, students’ perceptions were evaluated. Experimental interventions would be necessary to more accurately assess the real impact of these practices. In addition, the coping construct was operationalized exclusively through active strategies, as the instrument employed did not include maladaptive or avoidant responses. While this approach allowed the analysis to focus on functional resources, it may have reduced the model’s sensitivity to context-dependent differences in stress regulation.

Finally, because the sample was drawn from a single face-to-face university setting, the findings have limited generalizability across different cultural and institutional contexts. Moreover, the study did not collect more detailed demographic data (e.g., socioeconomic status, race/ethnicity, or first-generation student status), which constrains the ability to control for potential confounding variables and to explore moderating effects. Future research should include samples from diverse geographical regions and instructional modalities (e.g., online or hybrid formats), broaden the scope of good practices assessed, collect additional sociodemographic variables, and consider key contextual factors such as teaching climate and perceived institutional support. These enhancements would improve the external validity of the findings and increase their relevance and applicability across varied educational settings.

## 5. Conclusions

This study confirms that students’ good practices moderate the relationship between active coping and academic stress responses. Seeking feedback, engaging in cooperative work, and managing time efficiently progressively enhance the protective effect of coping. In contrast, active learning only amplifies this effect when paired with high levels of engagement and well-developed self-regulatory competencies. This differential pattern points to the need for tailored interventions: while the former practices can be fostered through relatively simple measures—such as formative feedback rubrics, early consultation forums, or brief time-management workshops—active learning requires more structured pathways and prior training in self-regulation.

Overall, the findings support an ecological view of academic coping, in which students’ good practices not only strengthen personal resources for stress management but also contribute to transforming academic demands into sustainable learning experiences. This perspective integrates individual, pedagogical, and organizational factors within a common framework of quality and student well-being. Accordingly, the systematic incorporation of these practices into curricula and institutional support programs is both justified and necessary. It should be noted, however, that this study is subject to certain limitations, including its cross-sectional design, non-probability cluster sampling, and reliance on self-reported data. To strengthen causal inferences and temporal understanding, future research should consider employing longitudinal or experimental designs.

## Figures and Tables

**Table 1:** Descriptive statistics and correlation matrix.

Variables	*M* (*SD*)	R-CEA	A-CEA	FS	CW	TM	AL
R-CEA	2.57 (0.74)	1					
A-CEA	2.82 (0.47)	−0.27 **	1				
FS	2.44 (0.73)	−0.27 **	0.07 *	1			
CW	3.62 (0.55)	−0.36 **	0.25 **	0.06	1		
TM	4.01 (0.56)	−0.41 **	0.19 **	0.01	0.19 **	1	
AL	2.94 (0.63)	0.23 **	0.44 **	0.02	0.34 **	0.33 **	1

Note. R-CEA = stress responses; A-CEA = active coping; FS = feedback-seeking; CW = cooperative work; TM = time management; AL = active learning; *M* = mean; *SD* = standard deviation; * *p* < 0.05, ** *p* < 0.01.

**Table 2:** Results of the moderation model: Feedback-seeking.

Variables	*B* (*SE*)	Beta	*t*	*p*	Lower CI	Upper CI
Gender	0.22 (0.05)	0.14	4.76	<0.001	0.12	0.32
Age	0.00 (0.01)	0.00	0.00	1.000	−0.02	0.02
A-CEA	−0.26 (0.11)	−0.17	−2.44	0.015	−0.48	−0.04
FS	−0.15 (0.07)	−0.15	−2.33	0.020	−0.23	−0.01
A-CEA × FS	−0.11 (0.05)	−0.38	−2.15	0.032	−0.21	−0.01
**Model Summary**						
*R*^2^ (%)	19.7					
Model	*F*(1008, 1013) = 21.72, *p* < 0.001					
Assumptions						
Normality	*p* = 0.258					
Independence	2.04					
Homocedasticicy	*p* = 0.721					

Note. *B*: unstandardized coefficients; *SE*: standard error; Beta: standardized coefficients; *R*^2^: coefficient of determination; A-CEA: active coping; FS: feedback-seeking; CI = 95% Confidence Interval.

**Table 3:** Results of the moderation model: Cooperative work.

Variables	*B* (*SE*)	Beta	*t*	*p*	Lower CI	Upper CI
Gender	0.22 (0.05)	0.14	4.66	<0.001	0.12	0.32
Age	0.00 (0.01)	0.00	0.00	1.000	−0.02	0.02
A-CEA	−0.37 (0.13)	−0.23	−2.83	0.005	−0.63	−0.11
CW	−0.21 (0.08)	−0.15	−2.52	0.012	−0.37	−0.05
A-CEA × CW	−0.17 (0.08)	−0.60	−2.08	0.038	−0.33	−0.01
**Model Summary**						
*R*^2^ (%)	18.5					
Model	*F*(1008, 1013) = 21.22, *p* < 0.001					
Assumptions						
Normality	*p* = 0.200					
Independence	2.08					
Homocedasticicy	*p* = 0.398					

Note. *B*: unstandardized coefficients; *SE*: standard error; Beta: standardized coefficients; *R*^2^: coefficient of determination; A-CEA: active coping; CW: cooperative work; CI = 95% Confidence Interval.

**Table 4:** Results of the moderation model: Time management.

Variables	*B* (*SE*)	Beta	*t*	*p*	Lower CI	Upper CI
Gender	0.27 (0.05)	0.17	5.66	<0.001	0.17	0.37
Age	0.00 (0.01)	0.00	−0.17	0.868	−0.02	0.02
A-CEA	−0.45 (0.14)	−0.28	−3.11	0.002	−0.72	−0.18
TM	−0.32 (0.12)	−0.24	−2.65	0.008	−0.56	−0.08
A-CEA × TM	−0.18 (0.08)	−0.67	−2.23	0.026	−0.34	−0.02
**Model Summary**						
*R*^2^ (%)	21.6					
Model	*F*(1008, 1013) = 26.43, *p* < 0.001					
Assumptions						
Normality	*p* = 0.207					
Independence	1.99					
Homocedasticicy	*p* = 0.537					

Note. *B*: unstandardized coefficients; *SE*: standard error; Beta: standardized coefficients; *R*^2^: coefficient of determination; A-CEA: active coping; TM: time management; CI = 95% Confidence Interval.

**Table 5:** Results of the moderation model: Active learning.

Variables	*B* (*SE*)	Beta	*t*	*p*	Lower CI	Upper CI
Gender	0.21 (0.05)	0.14	4.57	<0.001	0.11	0.31
Age	0.00 (0.01)	0.00	0.17	0.868	−0.02	0.02
A-CEA	−0.41 (0.11)	−0.26	−3.65	<0.001	−0.63	−0.19
AL	0.32 (0.11)	0.27	3.05	0.002	0.10	0.53
A-CEA × AL	−0.23 (0.10)	−0.78	−2.35	0.019	−0.43	−0.03
**Model Summary**						
*R*^2^ (%)	23.1					
Model	*F*(1008, 1013) = 22.55, *p* < 0.001					
Assumptions						
Normality	*p* = 0.200					
Independence	1.98					
Homocedasticicy	*p* = 0.641					

Note. *B*: unstandardized coefficients; *SE*: standard error; Beta: standardized coefficients; *R*^2^: coefficient of determination; A-CEA: active coping; AL: active learning; CI = 95% Confidence Interval.

## Data Availability

The data are available upon request to the corresponding author.
